# Slope–temperature faceting diagram for macrosteps at equilibrium

**DOI:** 10.1038/s41598-022-21309-x

**Published:** 2022-10-11

**Authors:** Noriko Akutsu, Yasuhiro Akutsu

**Affiliations:** 1 Faculty of Engineering, Osaka Electro-Communication University, Hatsu-cho, Neyagawa, Osaka 572-8530 Japan; 2 Department of Physics, Graduate School of Science, Osaka University, Machikaneyama-cho, Toyonaka, Osaka 560-0043 Japan

**Keywords:** Materials science, Materials for devices, Theory and computation, Nanoscience and technology, Statistical physics, thermodynamics and nonlinear dynamics

## Abstract

Faceting diagrams between surface slope and temperature are calculated numerically based on statistical mechanics for inclined surfaces between (001) and (111) surfaces at equilibrium. A lattice model is employed that includes point-contact-type step–step attractions from the quantum mechanical couplings between neighbouring steps. Comparing the obtained faceting diagrams with the phase diagram for step bunching proposed by Song and Mochrie for Si(113), the effective step–step attraction energy for Si(113) is approximately estimated to be 123 meV. The slope dependences of the mean height of the faceted macrosteps with a (111) side surface and that with a (001) side surface are calculated using the Monte Carlo method. The faceting diagrams can be used as a guide for controlling the assembling/disassembling of faceted macrosteps for designing new surface arrangements.

## Introduction

To meet the urgent need for energy efficiency to address global warming, it has become important to develop methods to reliably produce semiconductor materials with low energy consumption. In particular, III–V and II–VI compound semiconductors such as SiC and GaN are expected to be appropriate materials to meet this purpose^1,2^. However, macrostep formation or step bunching on crystal surfaces degrades the quality of crystals of these materials in the melt or during solution growth^1^. Extensive experimental studies have investigated methods to prevent macrostep formation, but achieving macrostep-free crystal growth is still difficult. Therefore, fundamental theoretical studies are required to control step assembling/disassembling on inclined surfaces.

Extensive studies have been made on macrostep instabilities for vapour growth or for molecular beam epitaxy (MBE)^3–5^. However, there have been few theoretical studies on macrostep instabilities at equilibrium. Cabrera and Coleman^6^ and Cabrera^7^ studied the relationship between the anisotropy of the surface free-energy density (surface tension) and the morphology of an inclined surface. They showed that the “type II” anisotropic shape of surface tension causes a macrostep on an inclined surface. However, they did not develop a microscopic model for macrostep instabilities.

**Figure 1:**
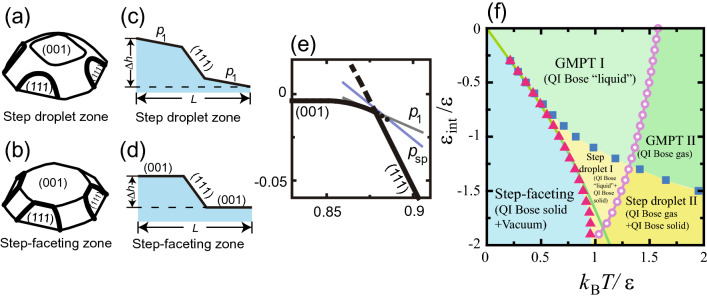
(**a**) and (**b**) Illustrations of perspective view of ECS (Andreev’s free energy) for step drop and step-faceting zones, respectively. Thin lines: facet edges without surface-slope *p* jumping. Thick lines: sharp edges of facets with *p* jumps (first-order shape transition^8,18^). (**c**) and (**d**) Side views of inclined surfaces at equilibrium based on results in Refs. 18 and 21. The slope *p* in the Monte Carlo method corresponds to $p=\Delta h/L$ with $\Delta h = N_\text{step}a$. (**e**) Cross section of ECS at $\langle 001 \rangle$–$\langle 111 \rangle$ plane in step droplet zone with $\varepsilon _\text{int}/\varepsilon = -0.9$ and $k_\text{B}T/\varepsilon = 0.63$. The surface slope *p* at $\eta$ is the slope of the tangential plane at $\eta$. $p_1$ represents the slope of the tilted surface at the coexistent point. $p_\text{sp}$ represents the slope of the surface at the spinodal point of the metastable surface. (**f**) $\varepsilon _\text{int}$-*T* faceting diagram^19,22^. The red triangles indicate $T_{f,2}$ values. The blue squares indicate $T_{f,1}$ values. The pink circles indicate the roughening transition temperatures for the (001) surface $T_\text{R}^{(001)}$. The green line is a zone-boundary line calculated by the 2D Ising model. The values for all the symbols were calculated using the PWFRG method. For definitions of the terms QI Bose solid, QI Bose liquid and QI Bose gas, please refer to Ref.^19^. This figure is taken from Ref.^19^.

Rottman and Wortis^8^ studied faceting transitions of the equilibrium crystal shape (ECS), i.e. the crystal droplet shape with the lowest total surface free energy^8–12^. The faceting transition is a phenomenon where a shrinking facet vanishes at a certain temperature on the ECS as the temperature increases. They adopted a three-dimensional (3D) Ising model with nearest neighbour (nn) and next nearest neighbour (nnn) couplings with anti-boundary conditions to form a 2D interface. They established that the faceting transition temperature is the same as the roughening transition temperature $T_\text{R}$ for the facet surface^8,13,14^. They also studied the case where the nnn coupling constant is anti-ferromagnetic, and showed that the ECS has a first-order shape transition at a facet edge (sharp facet edge) at low temperature (Fig. 1a,b,e). That is, the Wulff figure, the polar graph of the surface tension (surface free energy per normal area), becomes *discontinuous* at low temperature. As illustrated in Fig. 1e, the slope *p* of the tangential surface on the ECS is zero for the (001) surface, and the slope then increases continuously to $p_1$ as the surface point moves to the right. As this point moves further to the right, the slope of the surface jumps from $p_1$ to that for a (111) surface. Though they considered the shape transition, they did not provide details on the morphology of the inclined surface (Fig. 1c,d).

Williams and Bartelt^15^ observed a faceted macrostep on a Si(111) vicinal surface tilted towards [11-2]. The (111) terrace has a ($7\times 7$) structure, and the side surface of the macrostep is formed by a ($1\times 1$) structure. They considered macrostep formation based on two-surface coexistence caused by the surface free energy crossing for the ($7\times 7$) and ($1\times 1$) structures. With and without surface reconstruction, Jeong and Weeks showed that the faceted face spreads by a face-nucleation process^16^.

In our previous work^17,18^, we proposed a lattice model which shows step assembling/disassembling phenomena *at equilibrium* (Fig. 1c,d). The model is a restricted solid-on-solid (RSOS) model with point-contact-type step–step attractions (p-RSOS model, Eq. (1)). Here, “restricted” means that the height difference between nn sites is restricted to 0, ±1 (see the “Microscopic model” section). The point-contact-type step–step attractive energy $\varepsilon _\text{int}$ ($<0$) was introduced as a quantum mechanical interaction between elementary steps. The attractive energy is regarded as the energy gained by forming a bonding state by overlap between the electronic clouds of dangling bonds on neighbouring steps. It should be noted that point-contact-type step–step attractions do not exist for the inclined surface between the (001) and (101) surfaces in the p-RSOS model^18^. The adjacent steps on the inclined surface never occupy the same site at the same time. Hence, faceted macrosteps are not formed on the inclined surface between the (001) and (101) surfaces in the p-RSOS model.

The key property of the p-RSOS model is the discontinuous surface tension at low temperature^17–21^ for the inclined surface between the (001) and (111) surfaces. We found two transition points $T_{f,1}$ and $T_{f,2}$ for this inclined surface. For $T<T_{f,1}$, the surface tension is discontinuous around the (111) surface, while for $T<T_{f,2}$, the surface tension is discontinuous around the (001) surface. Corresponding to the connectivity of the surface tension, an $\varepsilon _\text{int}$–*T* faceting diagram was calculated^19,22^ (Fig. 1f). At sufficiently low temperature, $T<T_{f,2}$, we found a step-faceting zone where only the (001) and (111) surfaces are thermodynamically stable. The (001) facet edge in contact with the (111) surface becomes sharp (Fig. 1b), and two (001) surfaces and a (111) surface coexist to form an inclined surface (Fig. 1d). At sufficiently high temperatures, macrosteps almost disassemble, and we name this the GMPT zone after Gruber–Mullins–Pokrovsky–Talapov^23,24^. For $T<T_\text{R}^{(001)}$, where $T_\text{R}^{(001)}$ is the roughening transition temperature for the (001) surface, the ECS consists of (001) and (111) facets connected by a curved area (GMPT-I zone). The inclined surface consists of a series of elementary steps and locally merged steps.

Between the step-faceting and GMPT zones where $T_{f,2}<T<T_{f,1}$, we found a step droplet zone^17–21,25,26^. The (111) facet edge has a sharp edge (Fig. 1a). An inclined surface consists of a faceted (111) macrostep and tilted surfaces with a slope $p_1$ (Fig. 1c, step droplet I zone). Using the Monte Carlo method, we confirmed the classification of the structures on the inclined surface at equilibrium^21^. The $\varepsilon _\text{int}$–*T* faceting diagram is convenient for understanding the inclined surface structure qualitatively. However, details of the inclined structure in the step droplet zone cannot be derived from an $\varepsilon _\text{int}$–*T* faceting diagram.

The aim of this paper is to obtain the *p*–*T* faceting diagram for the step droplet zone using a Python re-coded product-wave-function renormalization-group (PWFRG) method^27–29^, which is a transfer matrix version of the tensor network^30^ method or the density matrix renormalization group (DMRG) method^31^. The slope dependence of the mean height of faceted macrosteps and faceted negative macrosteps is also calculated at equilibrium using the Monte Carlo method to see how the inhomogeneous structure (faceted macrosteps) is self-organised. In addition, we present a method to estimate the effective step–step attractive energy $\varepsilon _\text{int}$ approximately from experimental observations.

It should be noted that the step droplet zone does not exist in the terrace-step-kink (TSK) model^32^ with point-contact-type step–step attractions. The p-RSOS model is a more coarse grained model than the model used in the first principles quantum mechanical calculations^33^, whereas the p-RSOS model is a more microscopic model than the TSK model or phase-field model^34^. In the TSK model, excited structures contributing to the surface roughness, i.e., ad-atoms, ad-holes, islands, and negative islands (assembly of ad-holes), on the terrace surface are assumed to be irrelevant. However, recently, such excited structures were found to be relevant for fluctuating growing surfaces^35^. The appearance of the step droplet zone is one such surface-roughness relevant phenomenon. Hence, we focus on the effects of surface roughness and the diverse ways steps are gathered.

Since the 2D surface roughening transition belonging to the Kosterlitz–Thouless (KT) universality class^13^ is a rather subtle phenomena, more precise calculations are required^36^ than the mean-field or quasi-chemical calculations. Hence, to obtain reliable transition points, we used the PWFRG method to calculate surface free energies. In the PWFRG method, a dimensionality reduction and unit-padding are used iteratively^29^ to reduce the large number of atomic variables to a small number of “feature quantities”, as is done for deep learning in neural networks.

To study finite size effects for the step droplet zone, we adopt the Monte Carlo method, because the surface entropy in the finite-sized system is precisely taken into consideration. From the Monte Carlo simulations, we can see how the thermodynamic phenomena are smeared in the nano-scale size of the system. To compare the PWFRG results with the Monte Carlo simulations, long range step–step repulsion, which is known to exist in real-world systems such as elastic repulsion systems^37–39^, was not taken into consideration. Since we are studying equilibrium phenomena, we did not take various kinetic effects^3–5^ into consideration.

## Microscopic model

The surface energy of a (001) surface for the p-RSOS model^17,18^ is expressed by the following discrete Hamiltonian:

1 $$\begin{aligned}{\mathscr {H}}_\text{p-RSOS} &= {\mathscr {N}}E_\text{surf}+ \sum _{n,m} \varepsilon \left[ |h(n+1,m)-h(n,m)| +|h(n,m+1)-h(n,m)|\right] \nonumber \\&\quad +\sum _{n,m} \varepsilon _\text{int}\left[ \delta (|h(n+1,m+1)-h(n,m)|,2) +\delta (|h(n+1,m-1)-h(n,m)|,2)\right] , \end{aligned}$$

where *h*(*n*, *m*) is the surface height at site (*n*, *m*), ${{\mathscr {N}}}$ is the total number of lattice points, $E_\text{surf}$ is the surface energy per unit cell on the planar (001) surface, and $\varepsilon$ is the microscopic ledge energy for the nearest neighbour (nn) interactions. The summation with respect to (*n*, *m*) is taken over all sites on the square lattice. The RSOS condition on a square lattice, in which the height difference between the nn sites is restricted to $\{ 0, \pm 1\}$, is required implicitly. The fourth and fifth terms on the right-hand side of Eq. (1) represent the point-contact-type step–step attraction. $\delta (a,b)$ is the Kronecker delta and $\varepsilon _\text{int}$ is the microscopic point-contact-type step–step interaction energy. When $\varepsilon _\text{int}$ is negative, the step–step interaction becomes attractive (sticky steps).

The microscopic energies $\varepsilon$, $\varepsilon _\text{int}$, and $E_{\text{surf}}$ in the p-RSOS model (Eq. 1) are free energies from the viewpoint of first-principles quantum mechanical calculations. The surface energy $E_{\text{surf}}$ includes entropy originating from lattice vibrations and distortions^33^. $\varepsilon$ and $\varepsilon _\text{int}$ may decrease due to lattice vibrations as the temperature increases. However, they are assumed to be constant throughout this work.

It should be noted that the RSOS model is used to study the roughening transition^40–42^. The “RSOS model” used to study non-linear equations^43,44^ for fluctuating interfaces corresponds to the ASOS model for studying the roughening transition^40^.

## Results

### ${\varvec{p}}$–${\varvec{T}}$ faceting diagrams

#### Calculation of faceting diagram

We introduce the chemical potential for a step and calculate the grand partition function $\mathscr {Z}$^45–48^:

2 $$\begin{aligned} \mathscr {Z}(\vec {\eta }) =\sum _{\{h(m,n)\}}\exp [-(\mathscr {H}_\text{p-RSOS} -\Delta \vec {h} \vec {\eta }/a)/k_\text{B}T], \quad {\tilde{f}}(\vec {\eta }) = \lim _{\mathscr {N}\rightarrow \infty } -(k_\text{B}T/\mathscr {N} )\ln {\mathscr {Z}(\vec {\eta }) }, \end{aligned}$$

where $\vec {\Delta h}= (\Delta h_x, \Delta h_y) = (h(n+1,m)-h(n,m),h(n,m+1)-h(n,m)$ represents the height difference, $\vec {\eta }=(\eta _x,\eta _y)$ represents the chemical potential for a step, $a=1$ represents the lattice constant, and ${\tilde{f}}(\vec {\eta })$ represents Andreev’s free energy as a grand potential for the grand partition function. To calculate the grand partition function, we use the transfer matrix method. The grand potential is calculated based on the largest eigenvalue of the transfer matrix, where the PWFRG method is applied. It is known that ${\tilde{f}}(\vec {\eta })= \lambda z(-\lambda x, -\lambda y)$, where $\lambda$ is a Lagrange multiplier related to the volume of a crystal droplet, $\eta _x=-\lambda x$, and $\eta _y=-\lambda y$; that is, the shape as a function of $\vec {\eta }$ is similar to the ECS^8^.

The slope of the surface fluctuates under a given $\vec {\eta }$ at a temperature *T*. The surface gradient $\vec {p}=(p_x,p_y)$ is calculated as $(\langle \Delta h_x \rangle , \langle \Delta h_y \rangle )/a$. We designate $|\vec {p}|$ and $|\vec {\eta }|$ as *p* and $\eta$, respectively. The surface free energy $f(\vec {p})$ is calculated by $f(\vec {p})= {\tilde{f}}(\vec {\eta }) + \vec {\eta } \cdot \vec {p}$^47^. The surface tension $\gamma (\vec {p})$ is calculated by $f(\vec {p})/\sqrt{1+\vec {p}\cdot \vec {p}}$^49^.

The *p*–*T* and $\varepsilon _\text{int}$–*T* faceting diagrams are calculated using the following procedure. For a (111) surface, the surface free energy per projected *x*–*y* area is expressed by *f*(1, 1). Then Andreev’s free energy for the (111) surface is given by ${\tilde{f}}^{(111)}(\vec {\eta })=f(1,1)-\eta _x-\eta _y.$ ${\tilde{f}}(\vec {\eta })$, $\vec {p}(\vec {\eta })$, and ${\tilde{f}}^{(111)}(\vec {\eta })$ are directly calculated^46–48^ for each $\vec {\eta }$ using the PWFRG method. For a given temperature $T_{f}$ and $\varepsilon _\text{int}$, we calculate $\vec {p_1}$ from the two-surface coexistence condition (Fig. 1e):

3 $$\begin{aligned} {\tilde{f}}(\vec {\eta }^*)={\tilde{f}}^{(111)}(\vec {\eta }^*), \quad \vec {p_1} = \vec {p}(\vec {\eta }^*), \end{aligned}$$

where $\vec {\eta }^*=-\lambda (x^*,y^*)$ is $\vec {\eta }$ at the two-surface coexistent point.

#### Faceting diagrams

**Figure 2:**
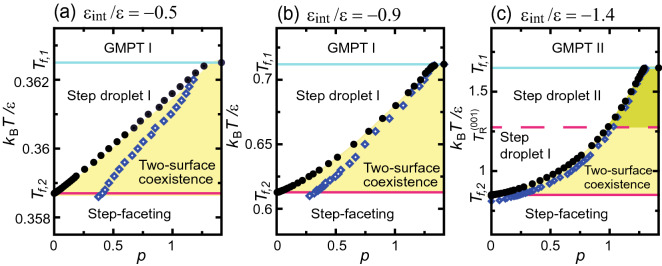
*p*–*T* faceting diagrams based on calculations using PWFRG (tensor network) method. Black filled circles: step assembling/disassembling points $(T_{f}, p_1)$. Open diamonds: spinodal points $(T_{f}, p_{sp})$ for homogeneous surfaces at temperature $T_{f}$. The errors are within the size of the data markers. $T_{f}$ converges to $T_{f,1}$ and $T_{f,2}$ for $p \rightarrow \sqrt{2}$ and $p \rightarrow 0$, respectively. For $T_{f,1}\le T$ (GMPT I or GMPT II), the inclined surface is homogeneous and consists of elementary steps and locally merged steps. For $T \le T_{f,2}$ (step-faceting zone), the inclined surface consists of (001) surfaces and a (111) surface. For $T_{f,2}<T< T_{f,1}$, the surface structure for $p_1<p$ is different from the structure for $p<p_1$, where $(T_{f}, p_1)$ is the first-order shape transition point. For $p<p_1$, the inclined surface consists of a homogeneous slope; whereas, for $p_1<p$, the inclined surface consists of a (111) surface and “terrace” surfaces with a slope $p_1$. For $p_1<p<p_\text{sp}$, the inclined surface can have a homogeneous slope *p* as a metastable surface. (**a**) $\varepsilon _\text{int}/ \varepsilon =-0.5$. (**b**) $\varepsilon _\text{int}/ \varepsilon =-0.9$. (**c**) $\varepsilon _\text{int}/ \varepsilon =-1.4$. Broken line: roughening transition of the (001) surface $T_R^{(001)}/\varepsilon = 1.28$, dividing the step droplet zone into two regions.

Figure 2 shows *p*–*T* faceting diagrams calculated by the PWFRG (tensor network) method. Figure 2a,b show the diagrams for $T<T_\text{R}^{(001)}$, while the diagram in (c) includes the temperature range $T_\text{R}^{(001)}<T$. The light blue and the red lines in the diagrams show the zone boundaries $T_{f,1}$ and $T_{f,2}$, respectively, and the red broken line in (c) shows $T_\text{R}^{(001)}$ for $\varepsilon _\text{int}/\varepsilon =1.4$. The black circles are the transition points calculated by the PWFRG method, and can be denoted by $(p_1, T_{f})$.

As seen from Fig. 2a, the phase separation points (black circles) are almost lined up to form the phase separation line for $\varepsilon _\text{int}/\varepsilon = -0.5$. On the other hand, the phase separation lines are concave for $\varepsilon _\text{int}/\varepsilon = -0.9$ and −1.4 in (b) and (c). In the step droplet I zone, at temperatures lower than the phase separation points, the inclined surface at (*p*, *T*) consists of a “stepped” surface with a slope $p_1$ ($<p$) at $T_{f}=T$ and a faceted macrostep with a (111) side surface (two-surface coexistence); whereas at temperatures higher than the phase separation points, the inclined surface consists of (001) terraces, elementary steps, and locally merged macrosteps with a (111) side surface. In the step droplet II zone (Fig. 2c), the (001) terraces and elementary steps are not well defined because the (001) surface is rough. However, the negative steps, which are (111) terraces and steps with (001) side surfaces, are well defined because the roughening temperature for a (111) surface in the RSOS model is infinite.

It appears that $p_1$ jumps at $T_{f,1}$ around $\sqrt{2}$. We consider that the jump occurs because of the terrace-step-kink (TSK) characteristics. The inclined surface near the (111) surface has a structure close to an ideal TSK due to the RSOS restriction^35^. We will return to this phenomenon in the “Discussion” section.

#### Spinodal points

In the step droplet zone, we can calculate surface tension for the metastable surface^17,50,51^, illustrated by the dotted line in Fig. 1e. The end of the dotted line, where the calculated slope in the PWFRG method jumps to $\sqrt{2}$ (the (111) surface) at $\vec {\eta }_\text{sp}$, approximately gives a spinodal point. We designate the slope at the spinodal point by $p_\text{sp}$. The slope at $\vec {\eta }_\text{sp}$ gives $p_\text{sp}= |\vec {p}(\vec {\eta }_\text{sp})|$.

In Fig. 2, $p_\text{sp}$ are shown by open blue diamonds. At a given *T*, in the region $p_1<p<p_\text{sp}$, an inclined surface can exist without a macrostep as a metastable structure. The metastable region is found to be wider for smaller $|\varepsilon _\text{int}|$. This metastable surface can appear when the surface is quenched from a high temperature $T_{f}(p)<T$ to a lower temperature $T'<T_{f}(p)$ with $p<p_{sp}(T')$. For the (111) surface, we obtained spinodal points where the slope jumps from $\sqrt{2}$ to $p^{(111)}_{sp}<p_1$ or 0 at $\eta ^{(111)}_{sp}<\eta ^*$ as $\eta$ decreases. Hence, for changing $\eta$, the change in slope *p* draws a hysteresis curve, as for a magnet. The magnetic field *H* corresponds to $\eta$, and the magnetisation *M* corresponds to *p*^19^.

As a summary of this subsection, we make the following conclusions. For $p<p_1(T)$ (the non-shaded area in Fig. 2), the inclined surface has a homogeneous structure. Qualitatively, a faceted macrostep tends to be formed under the following conditions: (1) large absolute value of the step–step attractive energy, (2) low temperature, (3) large slope of the inclined surface. For two-surface coexistence (the shaded area in Fig. 2) at a given *T*, (1) the “stepped” surface coexisting with the (111) surface has a slope $p_1(T)$; the faceted (111) surface plays the role of a step-reservoir to keep the slope of the stepped surface at $p_1(T)$; (2) when *T* is higher than the roughening temperature for the (001) surface, the (001) terrace is not well defined; however, the surface with slope $p_1(T)$ coexists with the (111) surface.

### Application of ${\varvec{p}}$–${\varvec{T}}$ faceting diagram to real surface

The phase diagram for Si(113) + Si(114) was determined by Song and Mochrie^52,53^ about three decades ago. Nevertheless, the value of the step–step attractive energy has not yet been determined and to the best of our knowledge, the $\varepsilon _\text{int}$–*T* and *p*–*T* faceting diagrams including surface roughness entropy for an inclined surface for realistic models have not been calculated. We can approximately estimate the step–step attractive energy by applying our results. In this subsection, we show how to estimate the step–step attractive energy by comparing the phase diagram for Si(113) + Si(114) with the present faceting diagram.

**Figure 3:**
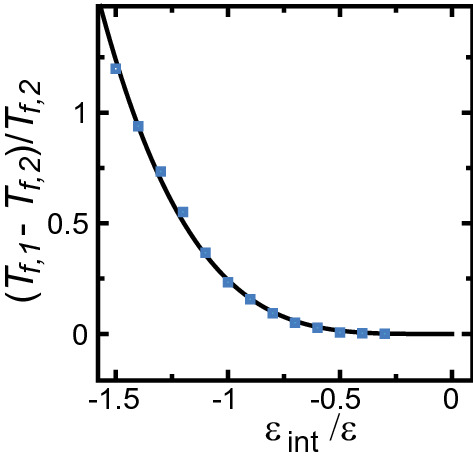
$\varepsilon _\text{int}/\varepsilon$ *vs* $(T_{f,1}-T_{f,2})/T_{f,2}$. Filled squares: PWFRG (DMRG) calculated points. Solid line: $y=0.244 x^4$, where $y=(T_{f,1}-T_{f,2})/T_{f,2}$, $x=\varepsilon _\text{int}/\varepsilon$.

The phase diagram for Si(113) + Si(114) determined by Song and Mochrie can be shown to be similar to the faceting diagram in the present study. To make this comparison, we plotted $\varepsilon _\text{int}/\varepsilon$ *vs* $(T_{f,1}-T_{f,2})/T_{f,2}$ in Fig. 3 from the $\varepsilon _\text{int}$–*T* and *p*–*T* diagrams. For Si(113), the inclined surface in the step-faceting zone, where $T_{f,2}=1134$K, consists of (113) and (114) surfaces, while the (113) faceted macrosteps disassemble at $T_{f,1} = 1223$K. Hence, $(T_{f,1}-T_{f,2})/T_{f,2} = 0.0785$. Then, using Fig. 3, we have $\varepsilon _\text{int} /\varepsilon = -0.753$. We finally have an approximate value of $\varepsilon _\text{int}=-123$ meV using the value of $\varepsilon = 163$ meV for Si(111) with the kink energy for $({\bar{1}}{\bar{1}}2)$^54^. In this manner, the microscopic effective step–step attractive energy can be approximately estimated by observing the step assembling/disassembling transition.

For those case where reliable $\varepsilon _\text{int}$–*T* and *p*–*T* faceting diagrams including surface roughness entropy for the objective materials have already been determined, a reliable value of $\varepsilon _\text{int}$ can be obtained using the present method. When $\varepsilon _\text{int}$–*T* or *p*–*T* faceting diagrams for an objective material do not exist, the faceting diagrams in the present study will help estimate $\varepsilon _\text{int}$. We address the reliability of estimated values of $\varepsilon _\text{int}$ in the “Discussion” section.

### Mean height of faceted macrosteps

#### Calculation of ${\varvec{\langle n \rangle }}$

To determine the structure of an inclined surface in a two-surface coexistent area including finite size effects, Monte Carlo simulations with the Metropolis algorithm were performed to calculate the mean height of the faceted macrosteps $\langle n \rangle$^22,25,26^ (Eq. 4). An inclined surface between a (001) surface and a (111) surface in the p-RSOS model is considered. Here, the temperature *T*, number of steps $N_\text{step}$, and system size *L* are fixed (external parameters). In contrast to the calculation of the surface free energy, the surface slope *p* is fixed and expressed by $p= N_\text{step}a/L$ with $a=1$. The energy of a surface configuration is given by Eq. (1). Atoms are captured from the ambient phase to the crystal surface and escape from the crystal surface to the ambient phase. To achieve equilibrium with the ambient (gas or solution) phase, the number of atoms in a crystal is not conserved. Several snapshots of top-down and side views of surfaces at $4 \times 10^8$ Monte Carlo steps per site (MCS/site) are shown in the supplementary information.

We introduce the ${\tilde{x}}$- and ${\tilde{y}}$-directions as the [110] and $[ {\bar{1}}10 ]$ directions, respectively. Here, ${\tilde{x}}$ is normal to the mean step-running direction and ${\tilde{y}}$ is along the mean step-running direction. When the height difference $\Delta h =1$ (or -1) at ${\tilde{y}}$ follows $n_{{\tilde{x}}}$ along the $+ {\tilde{x}}$ direction, we assign the height of a macrostep as $n_{{\tilde{x}}}({\tilde{y}})$ (or $- n_{{\tilde{x}}}({\tilde{y}})$). Then, the average macrostep height is obtained using the equation

4 $$\begin{aligned} \langle n \rangle =\sum _{{\tilde{y}}} \sum _{{\tilde{x}}}\left| n_{{\tilde{x}}}({\tilde{y}})\right| \Bigg / \left[ \sum _{{\tilde{y}}} n_\text{step}({\tilde{y}}) \right] \approx N_\text{step}/{\langle n_\text{step} \rangle }, \end{aligned}$$

where $N_\text{step}$ is the total number of elementary steps and $n_\text{step}({\tilde{y}})$ is the number of merged steps at ${\tilde{y}}$ along the ${\tilde{x}}$-direction. We take the time average of $\langle n \rangle$ over $2 \times 10^8$ MCS/site after the first $2 \times 10^8$ MCS/site are ignored. Further details of the Monte Carlo calculation method are given in Refs.^22,25^.

#### Step-faceting zone

**Figure 4:**
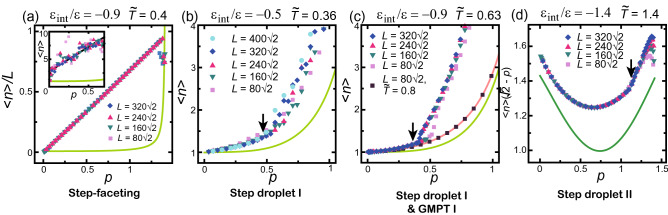
Slope dependence of $\langle n \rangle$. The initial configuration has a single macrostep. (**a**) Step-faceting zone. Inset: results obtained from the initial configuration with separated elementary steps at equal distances. (**b**) Step droplet I zone. (**c**) Step droplet I zone and GMPT-I zone (${\tilde{T}}= k_\text{B}T/\varepsilon = 0.8$). (**d**) Step droplet II zone. Arrows: (**b**) $p_1=0.4633$, (**c**) $p_1=0.3592$, (**d**) $p_1=1.100$. In (**b**)–(**d**), the results are independent of the initial configuration. $p=\Delta h/L = N_\text{step}a/L$. $a=1$. The average is taken over $2 \times 10^8$MCS/site after the first $2 \times 10^8$MCS/site are discarded. The light green lines in (**a**–**c**) show $\langle n \rangle$ for $\varepsilon _\text{int}=0$ at ${\tilde{T}}=0.4$ (Eq. 7). The dark green line in (**d**) is $\langle n \rangle$ for $\varepsilon _\text{int}=0$ at ${\tilde{T}}=1.4$ (Eq. 8). The orange line in (**c**) is $\langle n \rangle$ for $\varepsilon _\text{int}=-0.9$ at ${\tilde{T}}=0.8$ (GMPT-I) (Eq. 8).

For the step-faceting zone, Fig. 4a shows the *p* dependence of $\langle n \rangle /L$ where *L* is the size of the system. The inclined surface consists of (001) and (111) surfaces (Fig. 1b,d). Hence, $\langle n \rangle$ should be $N_\text{step}a=pL$ in the large size limit. The figure confirms the linear dependence of $\langle n \rangle /L$ on *p*. The slope of the line is slightly smaller than the expected value because the macrostep edges are smeared due to the finite size of the system^21^. In the limit $p \rightarrow \sqrt{2}$, the number of steps becomes $N_\text{step,max}-2$ to $N_\text{step,max}-4$ (2–4 negative steps^19^), where $N_\text{step,max}$ is the maximum number of steps, e.g. 640 for $L=320 \sqrt{2}$. Hence, the $\langle n \rangle /L$ values close to $p= \sqrt{2}$ in Fig. 4a lie off the line due to the finite-size effect.

In our previous work regarding the step-faceting zone in the non-equilibrium steady state^22,26,55^, we showed that the mean height of a faceted macrostep for $|\Delta \mu |< \Delta \mu _{co}(L)$ in the step-faceting zone is sensitive to the initial configuration. Here, $\Delta \mu$ represents the bulk chemical difference between the crystal and the ambient phases, $\Delta \mu _{co}(L)$ represents a crossover point between the 2D single nucleation and the poly-nucleation processes at the edge of the faceted macrosteps, and *L* is the linear size of the system. For $\Delta \mu _{co}< |\Delta \mu |$, $\langle n \rangle$ does not depend on the initial configuration. This indicate that the relaxation time from an initial configuration to reach the equilibrium configuration is longer than $2\times 10^8$MCS/site for $|\Delta \mu |< \Delta \mu _{co}$. Figure 4a shows the result obtained for an initial configuration with all the elementary steps merged. In the inset of the figure, we show the results starting from a configuration with all the elementary steps separated by an equal distance. The data are scattered around $\langle n \rangle (p) =2.80 + 8.87 p$. The mean values have only a small size dependence. For a separated-step initial configuration at low temperature, we obtained scattered results, where $\langle n \rangle (p) =2.08 + 5.19 p$ for $k_\text{B}T/\varepsilon = 0.2$, $\varepsilon _\text{int}/\varepsilon = -0.9$, and $L=80 \sqrt{2}$. Therefore, the mean height of local macrosteps is smaller than that for $k_\text{B}T/\varepsilon = 0.4$. This temperature dependence of the inclination of scattered data suggests how the surface achieves the equilibrium configuration without a 2D nucleation process. The zipping process of elementary steps^50,51,56^ is a candidate for this process. Clarifying this process is a subject for a future study.

#### Step droplet I zone or II

In the step droplet zone, $\langle n \rangle$ has a transition around a slope $p_1$, where $p_1$ is calculated by the PWFRG method, designated by an arrow in the figure. Figure 4c shows a typical example of Step droplet I zone. The obtained data do not depend on the initial configuration and are reproduced well. The first $2 \times 10^8$ MCS/site, whose data are ignored, was sufficient to achieve the equilibrium configuration. For $p<p_1$, $\langle n \rangle$ is close to the square marks and the orange line (Eq. 8), the results for the GMPT I ($k_\text{B}T/\varepsilon =0.8$). The obtained Monte Carlo data do not depend on the system size. This result indicates that the inclined surface in the un-shaded area in Fig. 2b has a homogeneous structure similar to that for the GMPT I zone. For $p_1<p$, $\langle n \rangle$ largely lie off the lines for the GMPT I and for $\varepsilon _\text{int}=0$. This is because a faceted macrostep is formed (Fig. S1). The transition point shifts to small *p* and converges to $p_1$ as the system size increases. This means that the elementary steps are difficult to assemble when the system size is small. If we assume *a* to be about 4Å, the calculated system size is about 40–200 nm. When the system size is less than 50 nm, the finite size effects become significant.

For $\varepsilon _\text{int}/\varepsilon =-0.5$ (Fig. 4b), the slope dependence of $\langle n \rangle$ for $p_1<p$ is different than that for large $\varepsilon _\text{int}$ systems in the step droplet zone. For $p_1<p$, the data fluctuate greatly and depend on the system size (Fig. S3). Considering a step-travelling time on the surface of longer than $2 \times 10^8$ MCS/site, we took an average over $4 \times 10^8$ MCS/site for $L=160 \sqrt{2}$. However, the fluctuation width hardly changed. The transition point is not clearly seen except for $L> 320 \sqrt{2}$. The (111) side surface can be easily separated when $\varepsilon _\text{int}$ is small. For $p<p_1$, $\langle n \rangle$ was close to the vales calculated at $k_\text{B}T/\varepsilon =0.63$. The obtained Monte Carlo data did not depend on the system size. Hence, in the un-shaded area in Fig. 2a also has a homogeneous structure similar to that in the GMPT I zone.

In the step droplet II zone (Fig. 4d), $p_1$ is large ($p_1=1.100$). Since the slope $\partial \langle n \rangle /\partial p$ is so steep, $\langle n \rangle (\sqrt{2}-p)$ is shown. Though the (001) surface is rough (Fig. S2), the behaviour of $\langle n \rangle$ is similar to that for the step droplet I zone. For $p<p_1$, $\langle n \rangle$ is larger than for $\varepsilon _{int}=0$, independent of the system size. This indicates that the inclined surface in the unshaded area in Fig. 2c has a homogeneous structure similar to that in the GMPT II zone. For $p>p_1$, $\langle n \rangle$ becomes larger than for the smaller system size.

#### Slope dependence of mean height of faceted negative macrosteps

**Figure 5:**
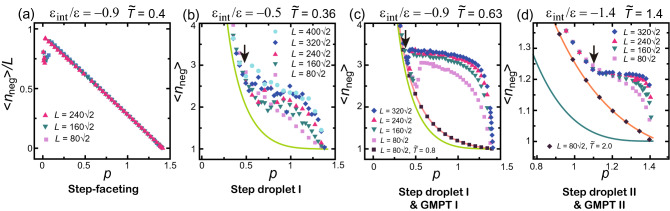
Slope dependence of the mean height of negative macrosteps $\langle n_\text{neg} \rangle =\langle n \rangle (\sqrt{2}-p)/p$^19^. Arrows: (**b**) $p_1=0.4633$, (c) $p_1=0.3592$, (**d**) $p_1=1.100$. The average is taken over $2 \times 10^8$MCS/site. (**a**) Step-faceting zone (${\tilde{T}}= k_\text{B}T/\varepsilon = 0.4$). (**b**) Step droplet I zone. (**c**) Step droplet I zone and GMPT-I zone (${\tilde{T}}= k_\text{B}T/\varepsilon = 0.8$). (**d**) Step droplet II zone and GMPT-II zone (${\tilde{T}}= 2.0$). The light green lines in (**b**) and (**c**) are $\langle n_\text{neg} \rangle$ for $\varepsilon _\text{int}=0$ at ${\tilde{T}}=0.4$ (Eq. 7). The dark green line in (**d**) is $\langle n_\text{neg} \rangle$ for $\varepsilon _\text{int}=0$ at ${\tilde{T}} =1.4$ (Eq. 8). The orange line in (**c**) is $\langle n_\text{neg} \rangle$ for $\varepsilon _\text{int}=-0.9$ at ${\tilde{T}}=0.8$ (GMPT-I) (Eq. 8). The orange line in (**d**) is $\langle n_\text{neg} \rangle$ for $\varepsilon _\text{int}=-1.4$ at ${\tilde{T}}=2.0$ (GMPT-II) (Eq. 11).

A negative macrostep has (111) terraces and a (001) side surface^19^. The mean height of negative macrosteps $\langle n_\text{neg}\rangle$ is obtained from the Monte Carlo results for $\langle n \rangle$ with $\langle n \rangle (\sqrt{2}-p)/p$^19^. $\langle n_\text{neg}\rangle$ are shown in Fig. 5.

In the step-faceting zone, $\langle n_\text{neg}\rangle$ scaled by *L* linearly decreases as *p* increases (Fig. 5a). In the limit $p \rightarrow 0$, the number of steps becomes 2–6. Hence, $\langle n \rangle /L$ values close to $p=0$ in Fig. 4a lie off the line due to the finite-size effect.

For the step droplet-I zone, Fig. 5c shows typical results. For $p<p_1$, $\langle n_\text{neg}\rangle$ is approximately the same as for the GMPT-I zone or the values on the line for $\varepsilon _\text{int}=0$. For $p_1<p$ in (c), $\langle n_\text{neg}\rangle$ converges to a constant value of 3.34 for $L =320 \sqrt{2}$. We designate this special value by $\langle n^*_\text{neg} \rangle$. Near $p=\sqrt{2}$, $\langle n_\text{neg}\rangle$ decreases sharply to one. Around $p \sim p_1$, $\langle n_\text{neg}\rangle$ for $p_1< p$ jumps from near the GMPT I line to a larger value. For small sizes, such as $L=80\sqrt{2}$, $\langle n_\text{neg}\rangle$ jumps from near the GMPT I line to a larger value, and gradually decreases as *p* increases. However, for large sizes, such as $L=320\sqrt{2}$, $\langle n_\text{neg}\rangle$ stays constant above $p_1$ as *p* increases with a small jump. These jumps in $\langle n_\text{neg}\rangle$ around $p \sim p_1$ are reproducible and do not depend on the initial configuration. Therefore, these jumps do not mean that the inclined surface is trapped in the metastable state. Rather, this jump in $\langle n_\text{neg}\rangle$ around $p \sim p_1$ is a characteristic of a system with a finite size at equilibrium. For the step droplet II zone (Fig. 5d), where $\sqrt{2}/2< p_1$, for $p<p_1$, $\langle n_\text{neg}\rangle$ is larger than for the GMPT-II zone. There are no jumps around $p=p_1$. For $p_1<p$, $\langle n_\text{neg}\rangle$ converges to $\langle n^*_\text{neg} \rangle = 1.22$ for a large system size.

When $\varepsilon _\text{int}$ (Fig. 5b) is small, the transition point $p_1$ is unclear except for $L> 320 \sqrt{2}$. $p_1 = 0.4633$ is calculated by the PWFRG method (the arrow in the figure). When $L= 400 \sqrt{2}$, the transition point agrees well with the value calculated by the PWFRG method.

In the two-surface coexistent state, the inclined surface with $p=N_\text{step}a/L$ is self-organised to form the (111) surface and stepped surfaces with a slope of about $p_1$. The (111) surface plays the role of a reservoir of elementary steps, keeping the stepped surface at $p_1$. When the (111) surface coexists with an inclined surface with $p_1$ for $p_1<p$, we obtain the following approximate equations for $\langle n \rangle$:

5 $$\begin{aligned} \langle n \rangle= & {} \frac{p}{p_1}\frac{(\sqrt{2}-p_1)}{(\sqrt{2}-p)(1-x)},\quad (p_1<1/\sqrt{2}), \nonumber \\ \langle n \rangle= & {} \frac{p}{(\sqrt{2}-p)(1-x)}, \quad (1/\sqrt{2} \le p_1<\sqrt{2}), \end{aligned}$$

and for $$\langle n_\text{neg} \rangle$$:

6 $$\begin{aligned} \langle n_\text{neg} \rangle= & {} \frac{(\sqrt{2}-p_1)}{p_1(1-x)},\quad (p_1<1/\sqrt{2}), \nonumber \\ \langle n_\text{neg} \rangle= & {} \frac{1}{(1-x)}, \quad (1/\sqrt{2} \le p_1<\sqrt{2}), \end{aligned}$$

where $1-x$ is a reduction factor, with *x* assumed to be 0, and $L \rightarrow \infty$. The key assumption is that all the elementary steps are separated, with $p_1$ on the surface coexisting with the (111) surface. $\langle n_\text{neg}^* \rangle$ corresponds to $\langle n_\text{neg} \rangle$ in Eq. (6). Qualitatively, Eqs. (5) and (6) describe the Monte Carlo results in Figs. 4 and 5 well.

Quantitatively, however, the values of $\langle n_\text{neg}^* \rangle$ for $p_1<p$ are larger than the values calculated by Eq. (6) with $x=0$. This is because locally merged steps decrease the number of steps $\langle n_\text{step} \rangle$ ( Eq.(4)). Taking $\langle n_\text{neg} \rangle$ in Eq. (6) to be equal to $\langle n_\text{neg}^* \rangle$ obtained by the Monte Carlo method, we obtained $x=0.3$, 0.13, and 0.05 for Fig. 5b–d, respectively. These values of *x* approximately equal the ratios of locally merged steps to elementary steps on an inclined surface with slope $p_1$.

## Discussion

The slope dependence of $\langle n_\text{neg}\rangle$ is not symmetric with $\langle n \rangle$ exchanging *p* by $(\sqrt{2}-p)$. The only difference in the inclined surface structure between $p=0$ and $p=\sqrt{2}$ is excited structures on the terrace such as ad-atoms, ad-holes, islands, and negative islands (clusters of ad-holes)^35^. As mentioned in the section “*p*-*T* faceting diagram”, the structure of an inclined surface sufficiently near the (111) surface of the RSOS model is close to the ideal TSK model due to the RSOS restriction.

In the ideal TSK model, the range of the attractive force is crucial. In the case of the point-contact-type step–step attraction, the configuration of the inclined surface should have all steps assembled or all disassembled. We can see this in the $\varepsilon _\text{int}$–*T* faceting diagram (Fig. 1f). For small $\varepsilon _\text{int}$, $T_{f,1}$ and $T_{f,2}$ become low. There are few excited structures on the (001) surface contributing to the surface roughness. The structure of the inclined surface near the (001) surface becomes close to the TSK model. This causes $T_{f,1}-T_{f,2}$ to be small, which we can see in Fig. 3. When the attractive force is short-range (longer than the point-contact-type), an inclined surface with slope $p_1$ can contact an inclined surface with slope $p_2$ ($<\sqrt{2}$), as was shown explicitly for the IC-RSOS model^47^. Such a structure has been observed on an inclined Si(111) surface^57^, where (7 $\times$ 7) and (1 $\times$ 1) surfaces compete.

In obtaining an approximate value of $\varepsilon _\text{int}$ for complex systems, as in a real material, the plausibility of the estimated value depends on how close the present model and the model for the materials are. For calculations of the p-RSOS model on a square lattice, the surface free energies and the faceting diagrams calculated by the PWFRG method in the present work are reliable. Note that the step droplet zone does not appear in the TSK model with the point-contact-type step–step attraction. It is crucial for the calculations of the faceting diagrams to take into consideration the surface roughness entropy. In this sense, we consider that details for a crystal such as the crystal structure are less relevant than the excited structures on the terrace surface. Even so, the calculations of the $\varepsilon _\text{int}$–*T* and *p*–*T* faceting diagrams using more detailed models will be considered in future studies.

Atomically rough–smooth surfaces have been proposed^58^, where an atomically rough surface indicates off-lattice deformations around the surface area. The structure has been found by molecular dynamical (MD) simulations^59^. Examples of atomically rough and thermodynamic smooth surfaces are $^4$He(0001) faceted crystal surfaces at a temperature below $T_\text{R}^{(0001)}$ in superfluid liquid He^60,61^, (011) faceted surfaces below $T_\text{R}^{(011)}$ for Ag$_2$S or Ag$_2$Se^62^, and (111) faceted surfaces below $T_\text{R}^{(111)}$ of Pb^63,64^ surrounded by vacuum. Examples of atomically rough and thermodynamic rough surfaces are the above surfaces at temperatures higher than $T_\text{R}$s, and liquid–vapor interfaces^65,66^. An atomically smooth surface is one in which the height of the surface $h(\vec {x})$ at a location $\vec {x}$ is well determined locally. Examples of atomically smooth and thermodynamic smooth surfaces are (001) surfaces described by lattice models at temperatures below $T_\text{R}$ and many clean flat semiconductor surfaces surrounded by vacuum. Examples of atomically smooth and thermodynamic rough surfaces are rough surfaces described by lattice models at temperatures higher than $T_\text{R}$^67^, and inclined surfaces described by lattice models as stepped surfaces at temperatures below $T_\text{R}$ for the terrace surface.

By definition, since the p-RSOS model is a lattice model, the surface of the model is atomically smooth. The atomically roughness entropy is not taken into consideration in the present work. However, the $\varepsilon _\text{int}$–*T* and *p*–*T* faceting diagrams may help estimate the effective $\varepsilon _\text{int}$ if the p-RSOS model is applied as a coarse-grained model in the same way as the TSK model. Systems with atomically rough surfaces have been treated using the roughening transition and faceting transitions obtained by lattice models. Both transitions belong to the KT universality class and exhibit GMPT universal behaviour at the facet edge on the ECS^23,24^. The details of the lattice structure are thought to be irrelevant for universal behaviour such as the Gaussian curvature jump at $T_\text{R}$^14^ and the Gaussian curvature jump at the facet edge at $T<T_\text{R}$^68^ on the ECS. When step tension reflecting the anisotropy with crystal symmetry is observable, this universal and non-universal behaviour can be studied experimentally. Nowicki et al.^64^ performed such observations on a Pb droplet with a mean diameter of 1 $\upmu$m and obtained a step–step repulsion of 16 meV Å$^{-2}$at about 350 K. To measure repulsive step–step interaction, the method using terrace width distribution function is known for vicinal surfaces^69–71^.

It should be noted that the faceting diagrams calculated by the PWFRG method are for systems in the thermodynamic limit ($L \rightarrow \infty$). To examine the finite-size effects, we performed Monte Carlo simulations. Assuming $a \sim$ 4Å, our results show system sizes of 45–200 nm. From the Monte Carlo results, the finiteness of the system size smears the singularity around $p_1$, especially for a size of less than 50 nm. A “crystal droplet” less than 50 nm in diameter cannot be regarded as a thermodynamic system. In a small-diameter droplet, the crystal structure is unstable. Similar to our results, a crystal droplet smaller than 50 nm, such as in the early stage of 3D nucleation, causes multi-step shape transformations. Multi-step shape transformations for growing crystal droplets have been observed for a Lennard–Jones system^72^, and Pt systems^73^ by MD simulations.

In our previous studies, the p-RSOS model was applied to non-equilibrium systems. We found zipping phenomena^50^ and pinning phenomena^51,56^. At a slope of $p=0.53$, we observed disassembling/assembling of faceted macrosteps^22,25,26^ and a faceted rough surface^55^ between the atomically rough and thermodynamic rough surfaces. From the slope dependence of the surface width, which is the standard deviation of the surface height, in the RSOS model ($\varepsilon _\text{int}=0$), the roughness exponent near (001) is different from that near (111) for large $\Delta \mu$^35^. The slope dependence of $\langle n \rangle$ and the surface width under non-equilibrium conditions is a problem for future studies.

## Conclusions

We calculated *p*–*T* faceting diagrams using the PWFRG (tensor network) method, shown in Fig. 2. We also calculated the mean height of faceted macrosteps $\langle n \rangle$ and faceted negative macrosteps $\langle n_\text{neg} \rangle$ using the Monte Carlo method shown in Figs. 4 and 5. From these results, we obtained the following conclusions.

- In the non-shaded area in the *p*–*T* faceting diagram where $p<p_1(T)$, the inclined surface has a homogeneous structure similar to the GMPT I or II zone.
- Qualitatively, a faceted macrostep tends to be formed under the following conditions: (1) large absolute value of the step–step attractive energy, (2) low temperatures, (3) large slope of the inclined surface, (4) large system size.
- For two-surface coexistence (the shaded area in Fig. 2) at a given *T* and $p_1(T)<p$ in the limit of $L \rightarrow \infty$: (1) the “stepped surface” has a slope $p_1(T)$ except for $p \approx \sqrt{2}$; the faceted (111) surface plays the role of a reservoir of elementary steps to keep the slope of the stepped surface at $p_1(T)$ in the step droplet I zone; (2) the mean heights of faceted macrosteps and faceted negative macrosteps are approximately given by Eqs. (5) and (6); (3) when *T* is higher than the roughening temperature of the (001) surface, the (001) terrace is not well defined; however, the surface coexisting with the (111) surface has a slope $p_1(T)$ except for $p \approx \sqrt{2}$; (4) when the system size is less than 50 nm, assuming *a* being about 4Å, the finite size effects become significant.
- For Si(113) + Si(114) surfaces, applying the results from the faceting diagrams Figs. 2 and 3, the effective step–step attractive energy $\varepsilon _\text{int}$ was approximately estimated to be $\varepsilon _\text{int}= -123$ meV.

## Methods

### Lines in Figs. 4 and 5

For $\varepsilon _\text{int}=0$ at ${\tilde{T}}=k_\text{B}T/\varepsilon =0.4$ and $L=80 \sqrt{2}$, it is known that the expressions $\langle n \rangle = s_1(p)/(\sqrt{2}-p)$ and $\langle n_\text{neg} \rangle = s_1(p)/p$ hold, where $s_1(p)$ is given by^35^

7 $$\begin{aligned} s_1(p) & = a_1 + a_2(p-b_1)^2 +a_4(p-b_1)^4, \nonumber \\ a_1&= 0.951, a_2=1.214, a_4=-0.604, b_1=0.707 . \end{aligned}$$

The lines are shown in light green in Figs. 4 and 5a–c. An extended form of Eq. (7) is $s_2(p)$

8 $$\begin{aligned} s_2(p)=\, c_1 + c_2(p-b_2)^2+c_3(p-b_2)^3+c_4(p-b_2)^4. \end{aligned}$$

For $\varepsilon _\text{int}=0$ at $k_\text{B}T/\varepsilon =1.4$, we have

9 $$\begin{aligned} c_1= 0.996, \ c_2=1.083, \ c_3=0.0638, \ c_4=-0.446, \ b_2=0.740. \end{aligned}$$

The lines Eq. (8) with Eq. (9) are shown in dark green in Figs. 4 and 5d. The orange lines in Figs. 4 and 5c represent the line for GMPT-I with $\varepsilon _\text{int}/\varepsilon =-0.9$ and at $k_\text{B}T/\varepsilon =0.8$ with $s_2(p)$, where

10 $$\begin{aligned} c_1= 1.129, \ c_2=0.597, \ c_3=0.00567, \ c_4=-0.1165, \ b_2=0.667. \end{aligned}$$

The orange line in Fig. 5d represents the line for GMPT-II with $\varepsilon _\text{int}/\varepsilon =-1.4$ and at $k_\text{B}T/\varepsilon =2.0$ with $s_2(p)$, where

11 $$\begin{aligned} c_1= 1.224, \ c_2=0.515, \ c_3=-0.0963, \ c_4=0.0626, \ b_2=0.757. \end{aligned}$$

## Supplementary Information

Supplementary Information.

## Data Availability

The datasets used and/or analyzed during the current study are available from the corresponding author on reasonable request.
