# Supplementary material for: Slope–temperature faceting diagram for macrosteps at equilibrium
Source: Sci Rep. 2022 Oct 11;12:17037. doi: 10.1038/s41598-022-21309-x (PMC9553894; doi:10.1038/s41598-022-21309-x)
Supplement: Supplementary file 1 — Supplementary Information. [file 41598_2022_21309_MOESM1_ESM.pdf]

Slope–Temperature Faceting Diagram for Macrosteps at Equilibrium

[1 *)]Noriko Akutsu, [2]Yasuhiro Akutsu
[1)] *Faculty of Engineering, Osaka Electro-Communication University, Hatsu-cho, Neyagawa, Osaka 572-8530, Japan*
[2)] *Department of Physics, Graduate School of Science, Osaka University, Machikaneyama-cho, Toyonaka, Osaka 560-0043, Japan*

[1*] nori3@phys.osakac.ac.jp

Images obtained by the Monte Carlo method

In this supplementary information, snapshots of surfaces obtained by the Monte Carlo simulation are shown at 4×10^8 Monte Carlo steps per site (MCS/site).

Fig. S1 presents top-down and side views of simulated surfaces for $\epsilon_{\rm int}/\epsilon = -0.9$ at the temperature $k_{\rm B}T/\epsilon = 0.63$. These are typical examples of vicinal surface structures in the step droplet zone I. When p is large enough, steps condensate to form a global macrostep with the side surface being the (111) surface.

Fig. S2 presents similar images to 1 for $\epsilon_{\rm int}/\epsilon = -1.4$ at the temperature $k_{\rm B}T/\epsilon = 1.4$. These are typical examples of vicinal surface structures in the step droplet zone II. Since the temperature is higher than the roughening temperature of the (001) surface ${T_{\rm R}}^{(001)}$, (001) "terraces" and elementary "steps" for vicinal surfaces with small p are not well defined, as shown in Fig. S2 (b).

For large $p > p_1$, the (111) terraces are well defined because the roughening temperature of the (111) surface ${T_{\rm R}}^{(111)}$ is infinite due to the RSOS restriction. The (111) surface layers are coloured red and blue, alternately. The border of the (111) terrace represents a negative step.

Fig. S3 presents similar images to 1 for $\epsilon_{\rm int}/\epsilon = -0.5$ at the temperature $k_{\rm B}T/\epsilon = 0.36$. All the images show examples for the step droplet zone I. However, since the step–step attraction is weak, steps do not condensate at a lower temperature than for a surface with $\epsilon_{\rm int}/\epsilon = -0.9$. The top-down view in Fig. S3 (b) is similar to 1 (b) but exhibits wider (001) terraces.

Significant smearing of the (111) faceted macrostep edges is seen in Fig. S3 (c). However, the (111) faceted macrostep edges are sharp for large system size, as shown in Fig. S3 (d), showing that the smearing of the (111) faceted macrostep edges is caused by the finite size effect.

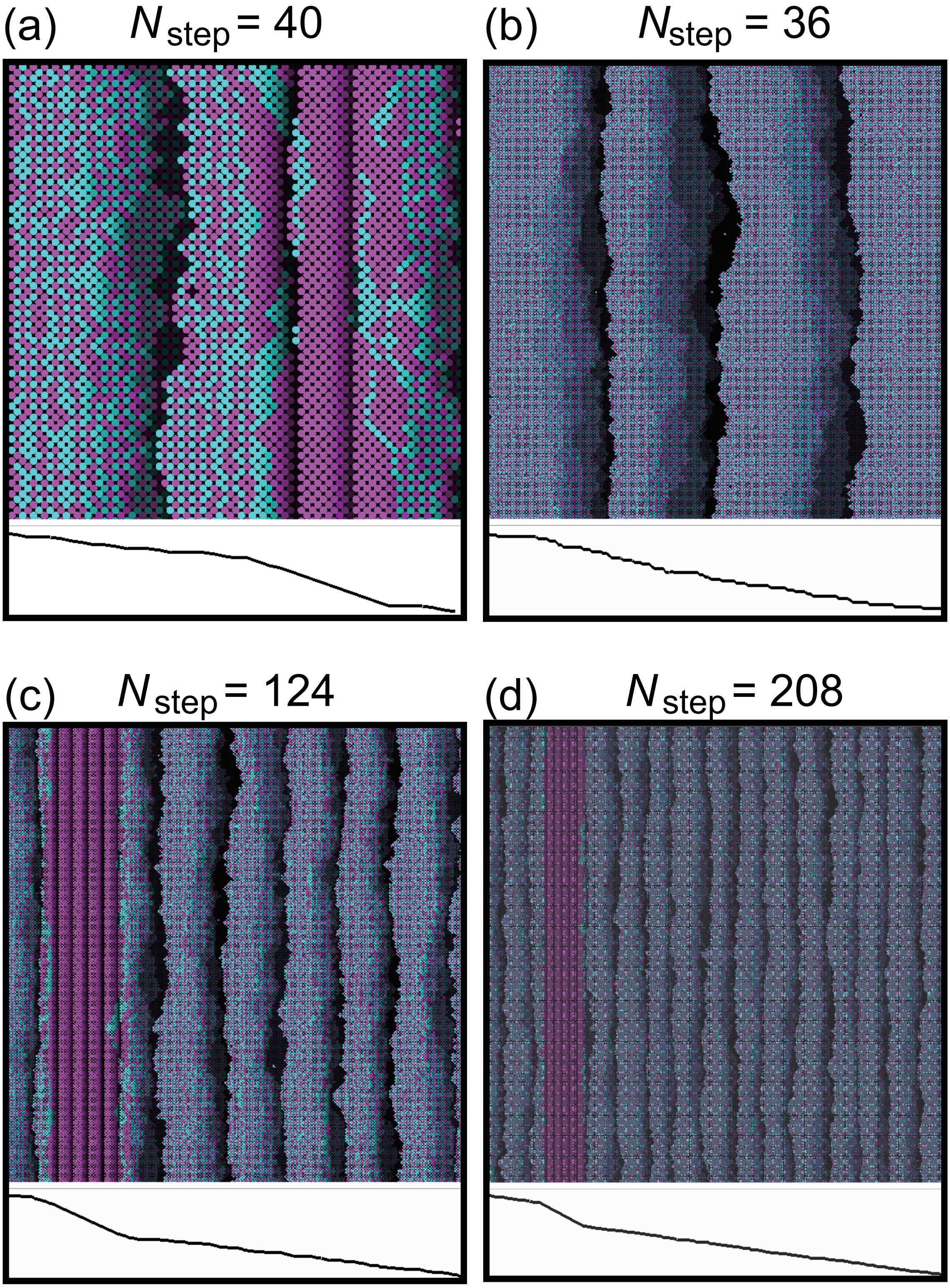

Figure S1. Snapshots of simulated surfaces at 4×10^8 MCS/site, with $\epsilon_{\text{int}}/\epsilon = -0.9$. $k_{\text{B}}T/\epsilon = 0.63$, showing top-down views (upper) and side views (lower). The height of the surface is expressed by the brightness with 10 repeated gradations. The (111) layers are coloured blue and red alternately. (a) $L = 40\sqrt{2}a$, $a = 1$, and $p = 0.7071$. (b) $L = 160\sqrt{2}a$ and $p = 0.1591$. (c) $L = 160\sqrt{2}a$ and $p = 0.5480$. (d) $L = 320\sqrt{2}a$ and $p = 4596$.

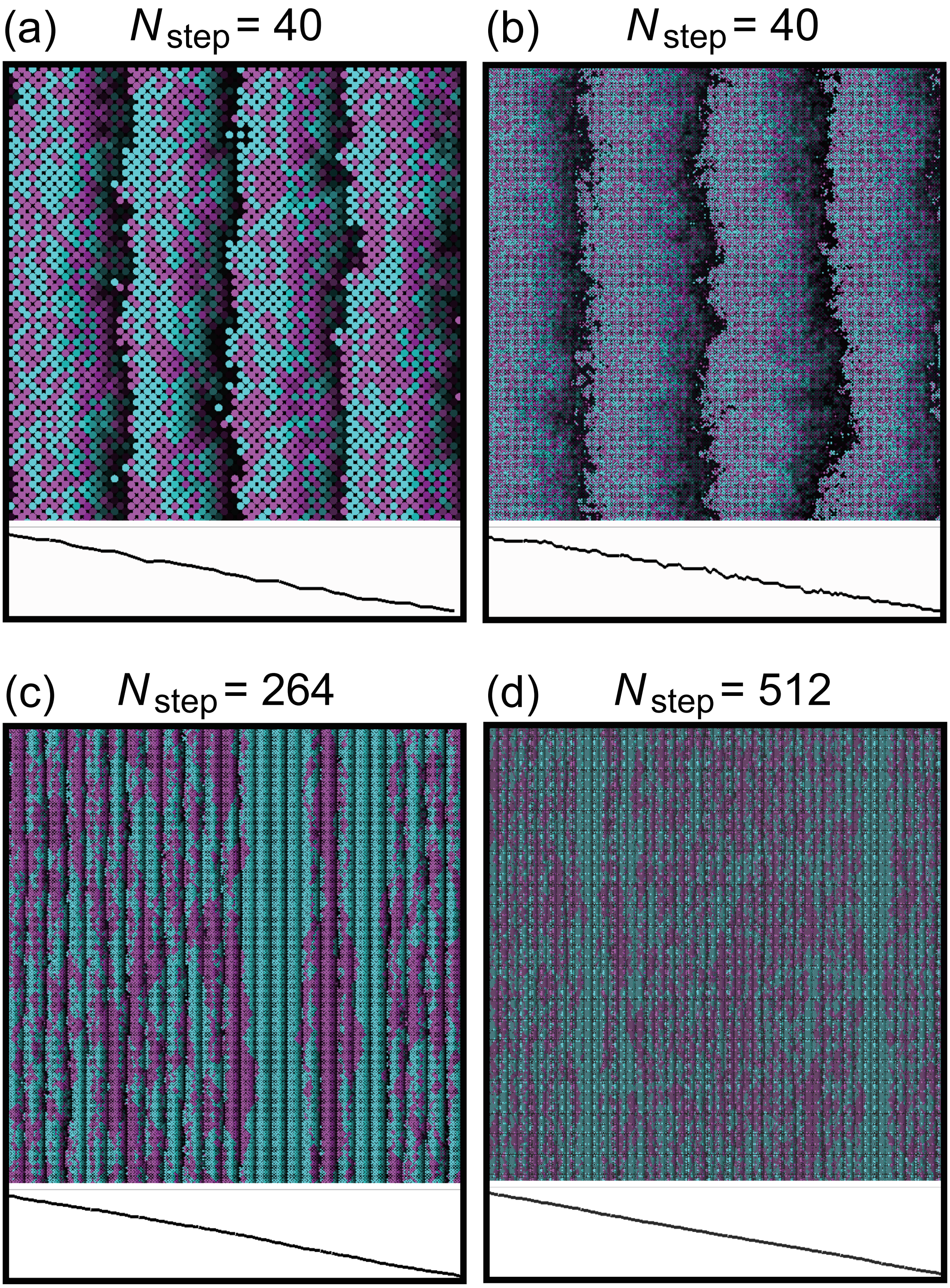

Figure S2. Snapshots of simulated surfaces at 4×10^8 MCS/site, with $\epsilon_{\mathrm{int}}/\epsilon = -1.4$. $k_{\mathrm{B}}T/\epsilon = 1.4$, showing top-down views (upper) and side views (lower). The height of the surface is expressed by the brightness with 10 repeated gradations. The (111) layers are coloured blue and red alternately. (a) $L = 40\sqrt{2}a$, $a = 1$, and $p = 0.7071$. (b) $L = 160\sqrt{2}a$ and $p = 0.1768$. (c) $L = 160\sqrt{2}a$ and $p = 1.167$. (d)$L = 320\sqrt{2}a$ and $p = 1.1314$.

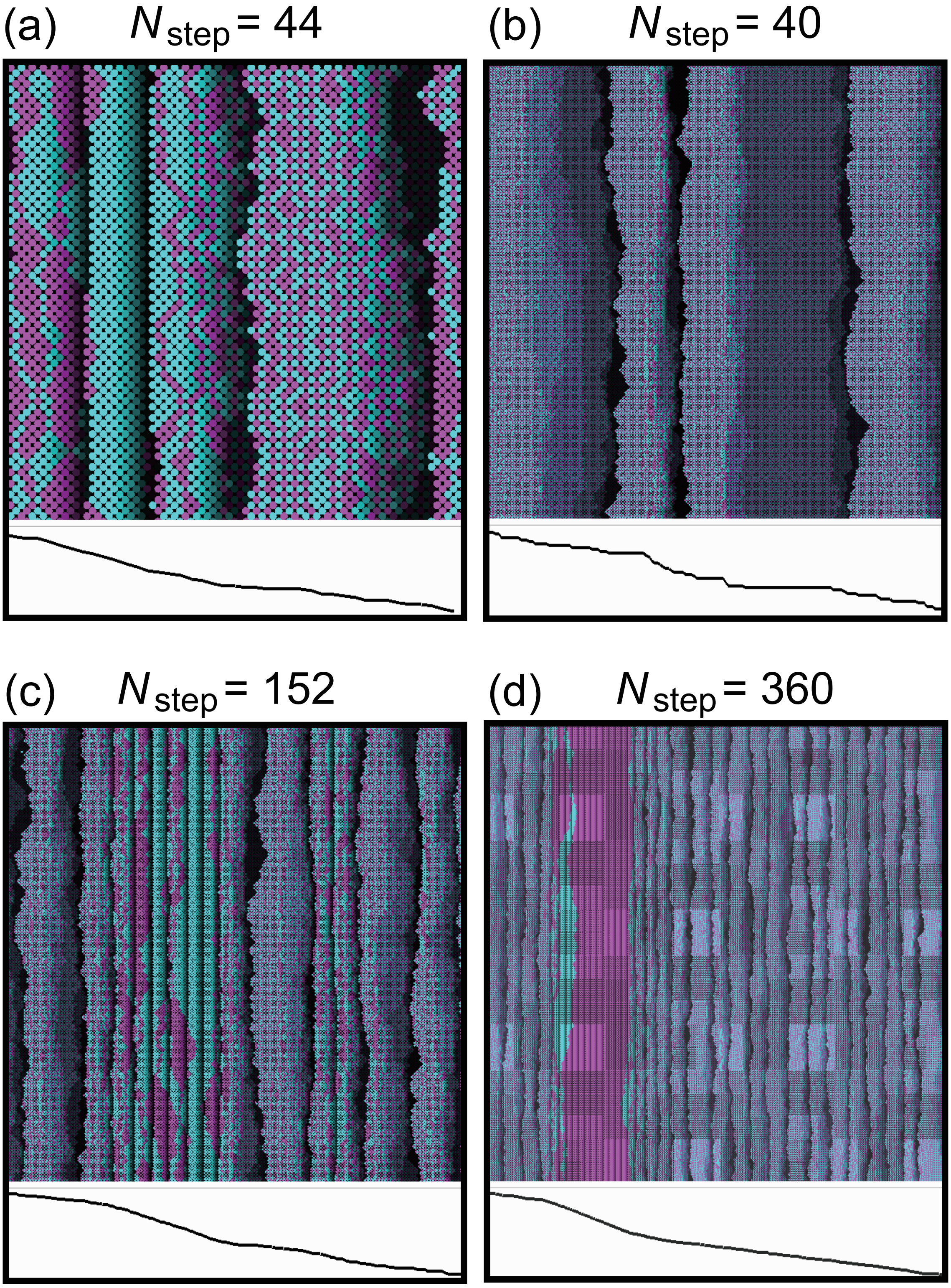

Figure S3. Snapshots of simulated surfaces at 4×10^8 MCS/site, with $\epsilon_{\mathrm{int}}/\epsilon = -0.5$. $k_{\mathrm{B}}T/\epsilon = 0.36$, showing top-down views (upper) and side views (lower). The height of the surface is expressed by the brightness with 10 repeated gradations. The (111) layers are coloured blue and red alternately. (a) $L = 40\sqrt{2}a$, $a = 1$, and $p = 0.7778$. (b) $L = 160\sqrt{2}a$ and $p = 0.1768$. (c) $L = 160\sqrt{2}a$ and $p = 0.6718$. (d)$L = 400\sqrt{2}a$ and $p = 0.6364$.
